# Reduction and functional outcome of open reduction plate fixation versus minimally invasive reduction with percutaneous screw fixation for displaced calcaneus fracture: a retrospective study

**DOI:** 10.1186/s13018-019-1162-5

**Published:** 2019-05-09

**Authors:** Ye Peng, Jianheng Liu, Gongzi Zhang, Xinran Ji, Wei Zhang, Lihai Zhang, Peifu Tang

**Affiliations:** Department of Orthopaedic Surgery, General Hospital of Chinese People’s Liberation Army, 28 Fu-Xing Road, Beijing, 100853 People’s Republic of China

**Keywords:** Minimally invasive, Calcaneus fracture, Percutaneous fixation

## Abstract

**Background:**

Minimally invasive surgery has become popular because of the lower incidence of wound complications. However, achieving an anatomic reduction that provides a satisfactory outcome is difficult using minimally invasive surgery. Our study aimed to evaluate the reduction and clinical outcomes of closed reduction and percutaneous fixation treatment using a closed reduction traction device for displaced intra-articular calcaneal fractures compared with traditional open reduction plate fixation using an extended lateral approach.

**Methods:**

A total of 40 patients and 45 feet with calcaneus fractures from 2012 to 2016 were studied. The open reduction plate fixation group (24 feet) was compared to the closed reduction percutaneous fixation group (21 feet) with a traction device. The reduction assessments included length, width, height, Bohler’s angle, Gissane’s angle, and varus or valgus angle before and after surgery. The clinical outcomes included the American Orthopaedic Foot and Ankle Society hindfoot score and the visual analog score for pain, length of stay, and complication rate.

**Results:**

The patients were followed up for an average of 16.53 ± 3.95 months. No significant differences in reduction were observed between the open and closed groups (*P* > 0.05). The American Orthopaedic Foot and Ankle Society scores of the two groups were 80.29 ± 6.15 and 83.62 ± 6.95 (open versus closed) (*P* = 0.0957). The visual analog scores of the open and closed groups were 1.50 ± 1.22 and 0.81 ± 0.87 (*P* = 0.0364). The lengths of stay in the open and closed groups were 9.63 ± 2.72 days and 6.71 ± 1.85 days (*P* = 0.0002). The complication rates of the open and closed groups were 20.8% (5/24) and 4.8% (1/21) (*P* < 0.0001).

**Conclusions:**

The closed reduction percutaneous fixation with traction device method may provide equivalent reduction results and superior outcomes for the length of stay, VAS score, and complication rate for displaced intra-articular calcaneal fractures.

## Introduction

Calcaneus fracture is a common fracture with an incidence of approximately 2% of all fractures and is the most common tarsal fracture (> 60%) [1]. Approximately 70% of calcaneus fractures are intra-articular fractures [2]. The gold standard treatment for intra-articular fractures is open reduction internal fixation using an extended lateral approach. However, many calcaneus fractures are associated with severe soft tissue injuries, which increase the risks of skin necrosis and infections, particularly in patients who smoke or have diabetes [3–5]. Surgeons have opted for less invasive techniques to reduce risks associated with performing the lateral extensile approach. For recent years, minimally invasive percutaneous fixation has become increasingly popular [6–8] because the minimally invasive method was associated with lower complication rates, shorter hospital stays, and more rapid healing [8, 9]. However, minimally invasive surgical techniques include limited exposure, which makes achieving a satisfactory closed reduction technically demanding and difficult to accomplish [10]. Many surgeons have questioned the quality of reductions achieved with minimally invasive techniques. In contrast, many open reduction surgeons have started to question the value of precise open reduction techniques because of the high rates of posttraumatic arthritis, chronic pain, postop fibrosis, and loss of subtalar function.

Many scholars have been working on the minimally invasive treatment of calcaneus fixations. Cao et al. [11] reported that a small lateral wall incision and minimally invasive plate fixation showed a good-to-excellent rate of 93.94% but did not report the wound complication rates. Battaglia et al. [12] and Dayton et al. [13] reported that with external fixation for calcaneus fractures, 18 of the 50 ankles (36%) developed arthritic changes in the lower ankle and 3 with sinus tarsi syndrome. Those results might be associated with the unsatisfactory reduction. None of them reported the reduction details or the results of reduction. Very few comparative studies have reported on open reduction plate fixation versus closed reduction percutaneous fixation and their functional outcomes.

**Fig. 1:**
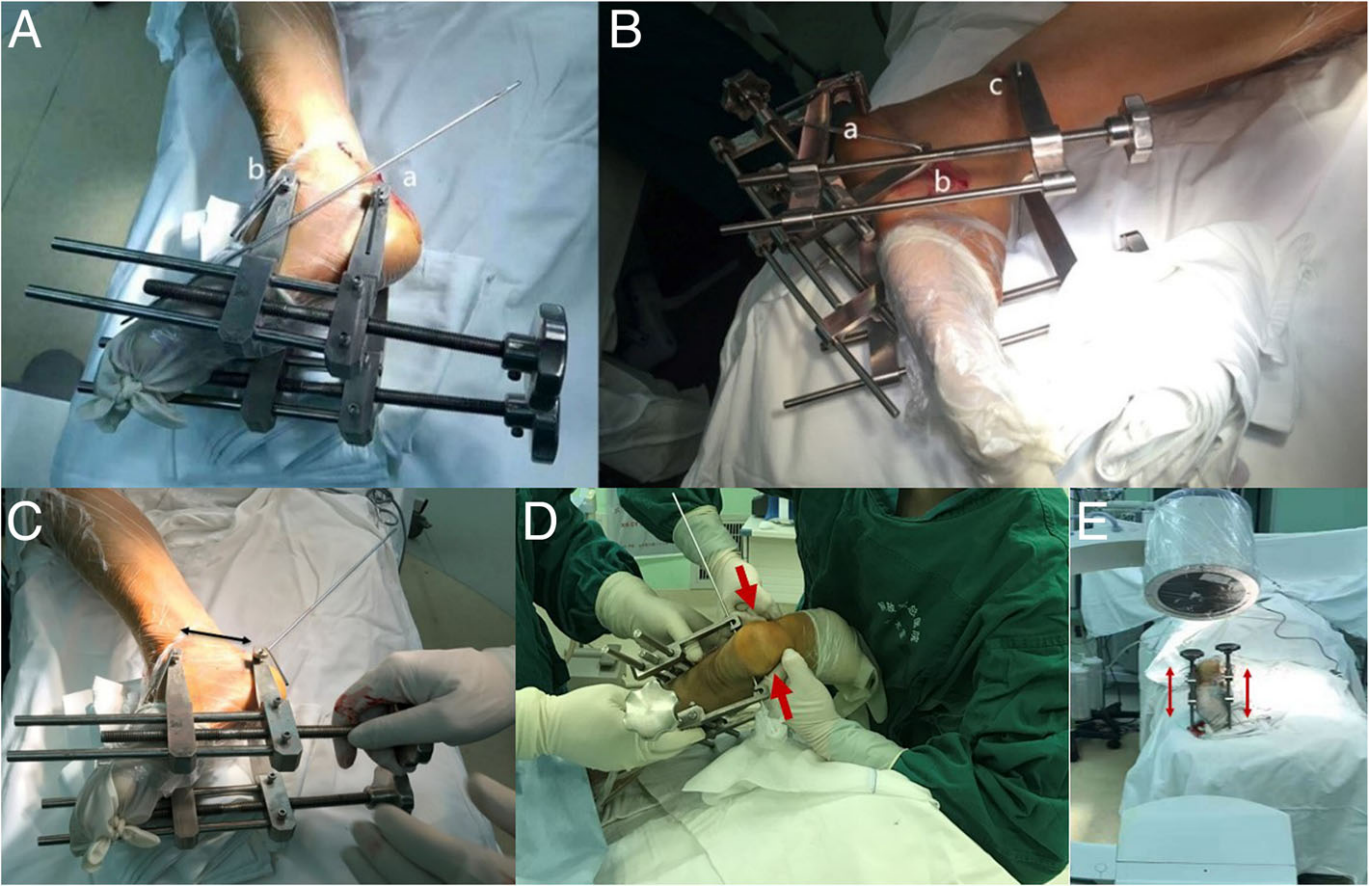
Two types of closed reduction devices and the closed reduction technique. **a** Two-point traction device (a: calcaneal tuberosity, b: talus neck). **b** Three-point traction device (a: calcaneal tuberosity, b: cuboid, c: tibia). **c** Axial traction for length reduction. **d** Compression by fists for width reduction. **e** Unilateral or bilateral traction for varus and valgus angle reduction

To address this problem, we need a minimally invasive method to achieve a precise reduction. Therefore, we fashioned two types of percutaneous closed reduction devices (Fig. 1) to provide traction for the subtalar joints. On the one hand, we can achieve a satisfactory reduction using a traction device and surgical technique. On the other hand, this reduction device is a closed reduction tool that will not affect the wound or increase the complication rate. After satisfactory reduction, percutaneous fixation can be accomplished according to standard procedures. In this study, we attempted to compare reductions via X-ray radiographs and AOFAS, VAS, complications of the short-term outcome and function of open reduction plate fixation versus closed reduction percutaneous fixation.

## Materials and methods

We retrospectively reviewed 40 cases of displaced intra-articular calcaneal fractures affecting 45 feet from June 2012 to December 2016. This study was approved by the People’s Liberation Army General Hospital Institutional Review Board. The hospital is a level 1 trauma center. The inclusion criterion was patients who underwent surgical treatment of a unilateral calcaneal intra-articular closed fracture without other associated fractures, and the exclusion criteria were patients who were treated conservatively and the lack of surgical conditions. The ICD-10 was used to identify the patients. In total, 23 patients were male, and 17 patients were female, with an average age of 43.7 years (range 18–67). The patients were separated into a closed reduction percutaneous group and an open reduction internal fixation group. The open reduction group operation was performed at 7–10 days after injury, after the swelling had subsided, and the closed reduction group operation was performed at 1–3 days after injury. The waiting days were accounted in the bed stay. All of the patients were discharged 2–5 days after surgery, depending on the situation of the wound. The surgery times of the two groups were recorded. A single senior orthopedic surgeon performed the surgeries. Smoking and diabetes status were recorded. X-rays and CT scans were obtained before the surgery. The VAS score was recorded on postoperative day 1 by the surgeon. The open reduction plate fixation group included calcaneus fractures of 24 feet: 11 of these were Sanders type II, 10 of these were Sanders type III, and 3 of these were Sanders type IV. The closed reduction percutaneous fixation group included calcaneus fractures of 21 feet: 12 of these were Sanders type II, 7 of these were Sanders type III, and 2 of these were Sanders type IV. The patient characteristics are shown in Table 1.

**Table 1:** Patient characteristics

	ORPF	CRPF
Gender	4 females/20 males	2 females/19 males
Age, years	39.2 (range, 16–65)	40.8 (range, 23–60)
Smoker, *n* (%)	7/24 (29.2%)	5/21 (24%)
Diabetes, *n* (%)	2/24 (8.3%)	1/21 (4.7%)
Sanders classification	11 type II/10 type III/3 type IV	12 type II/7 type III/2 type IV
Surgery time	87.00 ± 9.78 min	85.86 ± 11.17 min

Lateral and axial X-ray views were obtained before the surgery, and the length, width, height, Bohler’s angle, Gissane’s angle, and varus or valgus angle of the calcaneus were recorded before and after the surgery according to these views. The measurement method used was in accordance with a previous study [14]. The varus angle was defined as a positive value, and the valgus angle as a negative value. The measurements obtained from the X-ray images were determined by a senior attending orthopedic surgeon. The AOFAS hindfoot score was utilized for clinical outcome assessment. The AOFAS hindfoot score was recorded at the final follow-up, whereas the visual analog scores for pain were recorded after surgery. The length of stay was recorded during the hospitalization, and the complication rate was recorded separately. The complications included early complications (i.e., superficial infections, deep infections, and wound necrosis) and late complications (i.e., posttraumatic arthritis, stiffness, chronic pain, fixation failure, lateral impingement, and joint penetration).

### Implants

**Fig. 2:**
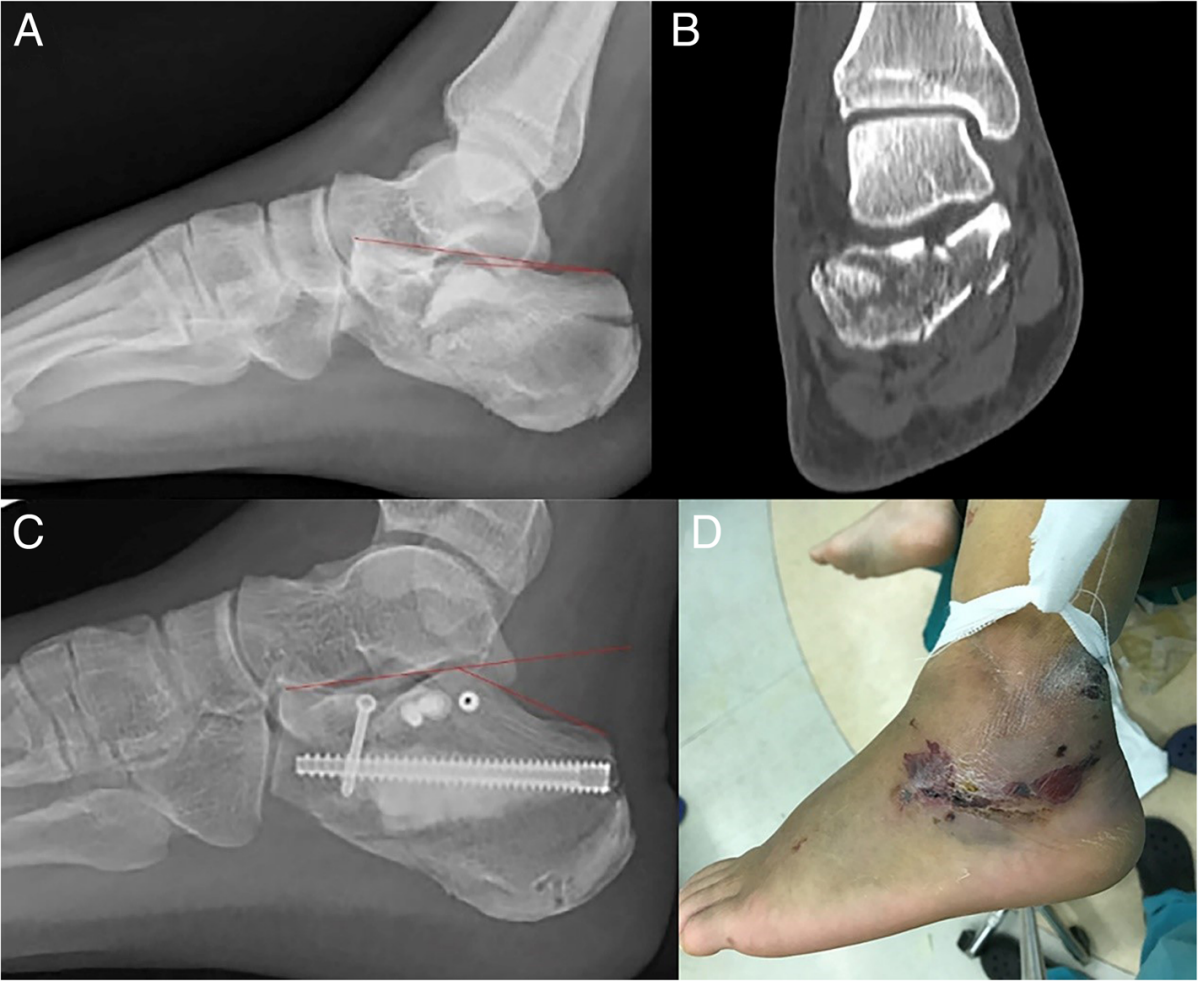
Case 1: A 40-year-old male with diabetes and severe soft tissue injury. X-ray (**a**, **c**) showing the lateral view of the calcaneus fracture before and after closed reduction percutaneous fixation surgery. The Bohler’s angle (red line) was reduced from − 3 to 28°. CT scan (**b**) showing a comminuted fracture in the articular surface (Sanders type IV). Bad soft tissue situation of calcaneous skin (**d**)

**Fig. 3:**
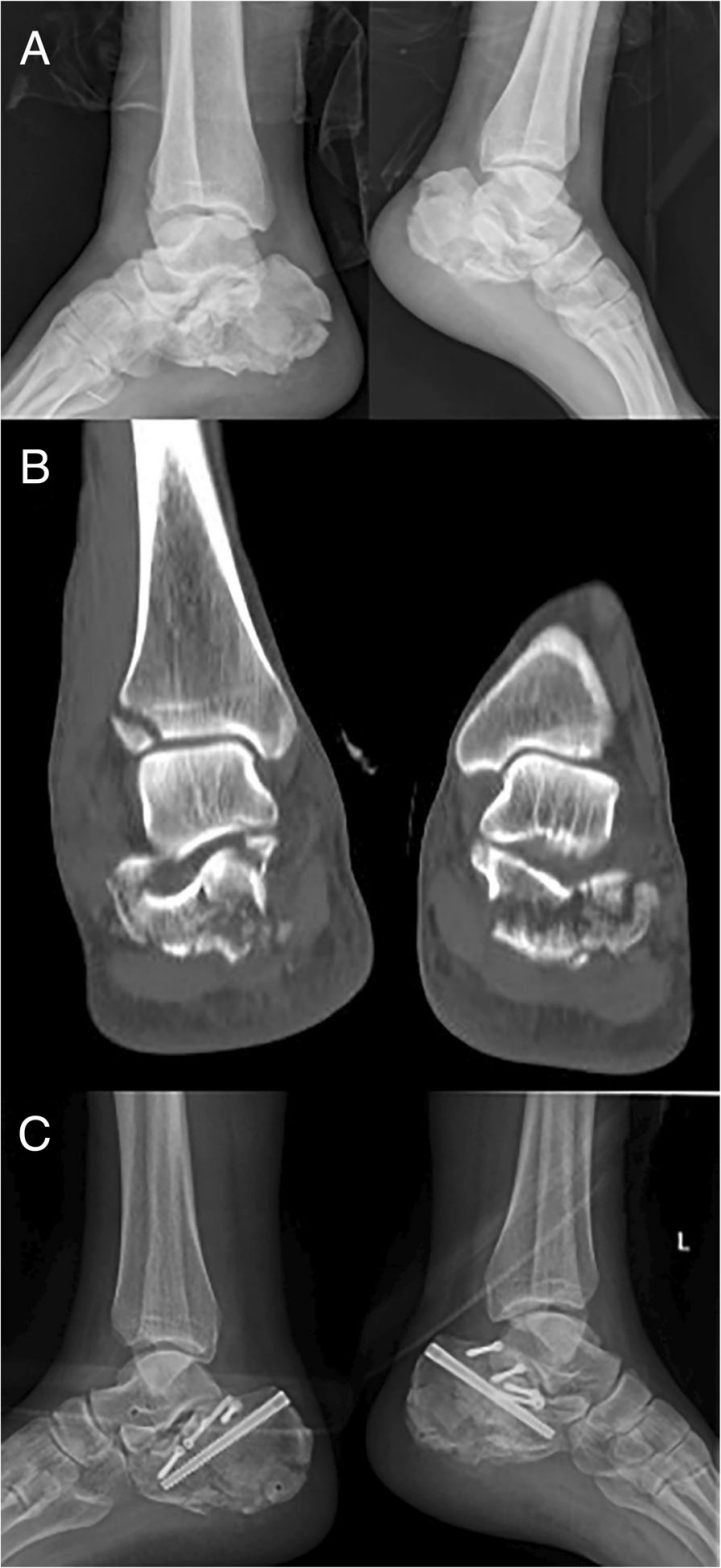
Case 2: A 38-year-old male who was a smoker with bilateral calcaneus fractures. X-ray lateral view (**a**) and CT scan (**b**) showing severe compression of the subtalar joint articular surface. Lateral view (**c**) after surgery showing satisfactory reduction and percutaneous fixations of the calcaneus fracture

The calcaneus locking plate system was selected for open reduction plate fixation. A 4.0-mm-diameter cannulated screw and a 7.0-mm-diameter cannulated screw and a high-strength injectable calcium phosphate graft were selected for closed reduction percutaneous fixation. A 7.0-mm-diameter cannulated screw was also implanted and removed to create a bone tunnel for reduction (Figs. 2 and 3).

### Standard operative technique

**Fig. 4:**
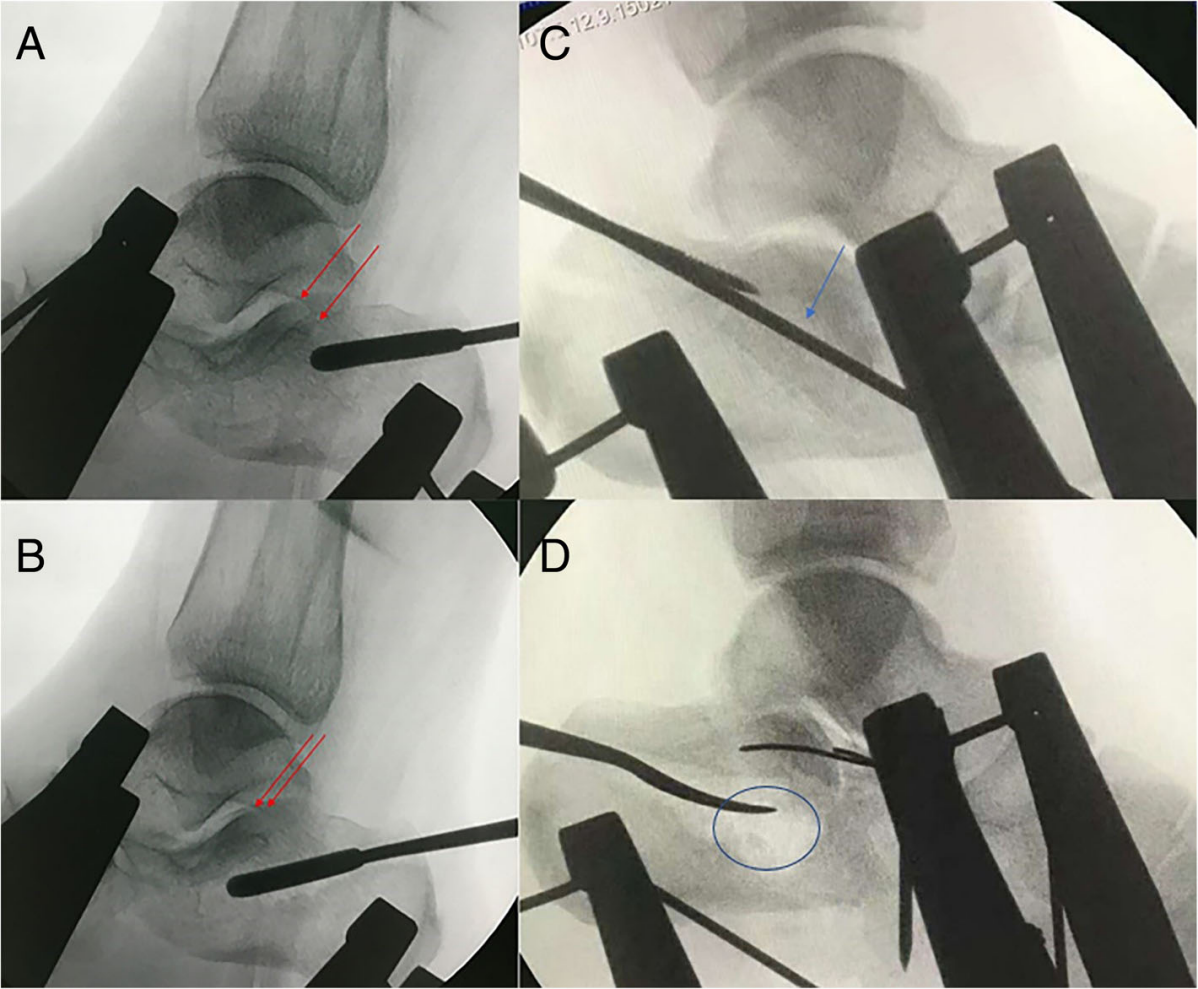
**a**–**d** Reduction of the depressed articular surface through the created bone tunnel with a curved smooth periosteal elevator. Before reduction (**a**). After reduction (**b**). Subtalar joint articular surface (red arrow). Depressed subtalar joint articular surface (blue arrow). Large bone defect after reduction (blue circle). Before reduction (**c**). After reduction (**d**)

**Fig. 5:**
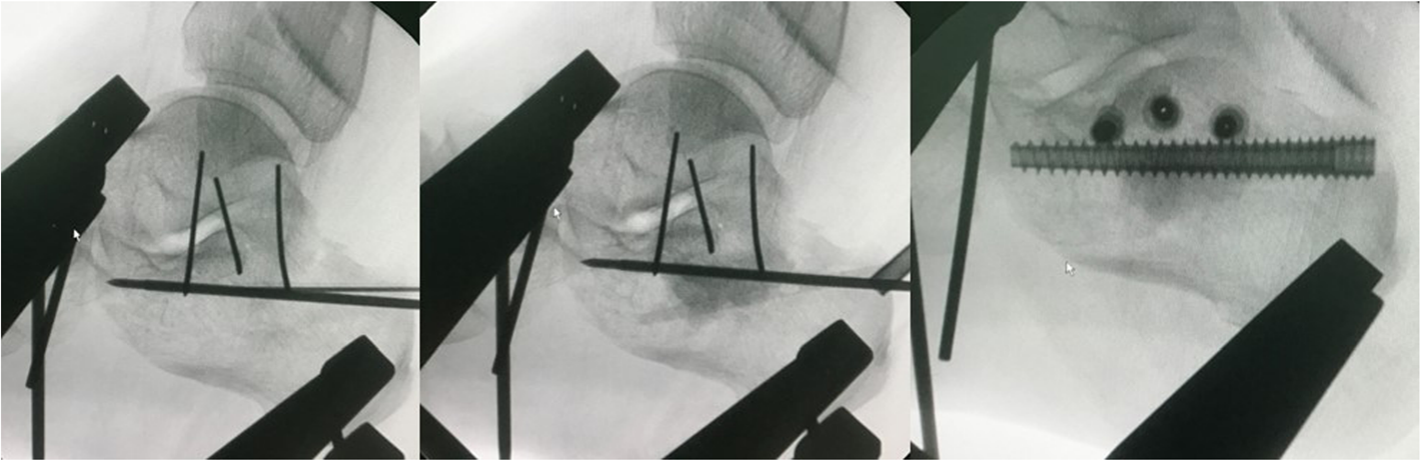
Calcium phosphate cement was injected through the bone tunnel to fill the bone defect space and increase the primary stability

The extensile lateral approach used was similar to the previously referenced technique [15]. For closed reduction percutaneous fixation, the patient was positioned in the prone position. After surgical draping, a localization K-wire on the skin was positioned perpendicular to the subtalar joint on the lateral view. Two K-wires were separately placed to penetrate perpendicularly to the calcaneus axis in the talus neck and into the calcaneus tuberosity. In some more comminuted cases (such as Sanders type IV), the front traction K-wire should be implanted in both the tibia and the navicular bone instead of the talus bone. Subsequently, we assembled the traction device on both sides of the calcaneus and gradually initiated traction with the pair of devices simultaneously. The traction was monitored by lateral views to confirm the subtalar joint was distracted. The length of the calcaneus was reduced, and the presence of the subtalar joint gap to elevate the compressed articular surface and lateral wall was verified. Then, an axial view of the calcaneus was obtained to permit calcaneus varus and valgus adjustment by retracting the unilateral or bilateral traction device. Additionally, the width was reduced by compression on both sides of the calcaneus applied by the assistant’s fists as monitored on the axial view. Next, an elevator bone tunnel could be established near the Achilles tendon attachment to inferior of the compressed subtalar joint by implanting and then removing the 7.0-mm cannulated screw from posterior to anterior. Through the bone tunnel, a smooth periosteal elevator may be used to elevate the central compressed articular surface under lateral view monitoring (Fig. 4). Through the established tunnel, the lateral and central surfaces of the subtalar joint may be reduced and temporarily fixed using a 4.0-mm cannulated screw guide wire percutaneously. When subtalar joint reductions were completed and assessed by lateral and axial views, a 4.0-mm cannulated screw was implanted for subtalar articular fixation. Then, calcium phosphate cement was injected through the tunnel to the bone defect space, and a 7.0-mm headless cannulated screw was fixed through the reduction tunnel along the calcaneal axis (Fig. 5). Finally, the wound was closed with sutures, and a bandage was applied.

### Postoperative management

Full exercise of the foot in bed was permitted the next day. Non-weight-bearing ambulation was begun from the second day until 4–6 weeks. After 4 weeks, lateral and axial view X-rays were obtained. Weight-bearing from a tolerated to full weight-bearing was permitted at 6–8 weeks after surgery. After 8 weeks, the patients were allowed to return to light work (after 10 weeks for medium work and 12 weeks for heavy work). All of the patients were followed up for more than 1 year (average 16.53 ± 3.95 months (range, 12 to 27)).

### Statistical analysis

Paired Student’s *T* tests were used to assess the reduction, length of stay, and scores for the different treatment groups. Chi-square tests were applied for complication rates. Statistical significance was defined as *P* < 0.05. The SPSS statistical software package for Windows (22.0) was used for the statistical analysis.

## Results

### Reduction assessment

Postoperatively, lateral and axial views were obtained to assess the reduction. We analyzed length, width, height, varus, and valgus angles for the calcaneus morphology reduction, and Bohler’s and Gissane’s angles for articular surface reduction.

The open reduction plate fixation group length was restored from 7.96 ± 0.70 (range, 6.38 to 9.05) cm preoperatively to 7.48 ± 0.61 (range, 6.48 to 8.43) cm postoperatively. The reduction percutaneous fixation group length was restored from 8.52 ± 0.80 (range, 7.20 to 9.79) cm preoperatively to 7.91 ± 0.69 (range, 6.79 to 8.93) cm postoperatively.

The open reduction plate fixation group width was restored from 4.60 ± 0.48 (range, 3.20 to 6.40) cm preoperatively to 3.93 ± 0.46 (range, 3.11 to 4.92) cm postoperatively. The reduction percutaneous fixation group width was restored from 4.24 ± 0.52 (range, 3.24 to 4.99) cm preoperatively to 3.84 ± 0.48 (range, 3.11 to 4.57) cm postoperatively.

The open reduction plate fixation group height was restored from 4.05 ± 0.59 (range, 3.40 to 4.79) cm preoperatively to 4.09 ± 0.51 (range, 3.19 to 4.72) cm postoperatively. The reduction percutaneous fixation group height was restored from 4.05 ± 0.36 (range, 3.40 to 4.55) cm preoperatively to 4.21 ± 0.56 (range, 3.35 to 4.83) cm postoperatively.

The open reduction plate fixation group Bohler’s angle was restored from 1.60 ± 25.94° (range, − 44.00 to 35.50°) preoperatively to 19.97 ± 9.48° (range, 5.90 to 41.00°) postoperatively. The reduction percutaneous fixation group Bohler’s angle was restored from 1.63 ± 13.55° (range, − 13.11 to 38.70°) preoperatively to 21.70 ± 10.33° (range, 11.7 to 47.3°) postoperatively.

The open reduction plate fixation group Gissane’s angle was restored from 118.57 ± 12.21° (range, 100.20 to 144.20°) preoperatively to 116.51 ± 10.61° (range, 101.7 to 140.00°) postoperatively. The reduction percutaneous fixation group Gissane’s angle was restored from 122.10 ± 14.95° (range, 84.40 to 135.50°) preoperatively to 120.18 ± 16.76° (range, 91.40 to 148.10°) postoperatively.

The open reduction plate fixation group varus or valgus angle was restored from 1.81 ± 4.20° (range, − 8.00 to 11.00°) preoperatively to 1.52 ± 2.35° (range, − 2.00 to 8.00°) postoperatively. The reduction percutaneous fixation group Gissane’s angle was restored from 3.30 ± 5.17° (range, − 8.00 to 13.00°) preoperatively to 1.41 ± 1.59° (range, − 1.00 to 4.00°) postoperatively.

The reduction measurements are shown in Table 2.

**Table 2:** Reduction radiographic parameters

	ORPF	CRPF	*P*
Length before surgery	7.96 ± 0.70 (cm)	8.52 ± 0.80 (cm)	–
Length after surgery	7.48 ± 0.61 (cm)	7.91 ± 0.69 (cm)	0.8764
Width before surgery	4.60 ± 0.48 (cm)	4.24 ± 0.52 (cm)	–
Width after surgery	3.93 ± 0.46 (cm)	3.84 ± 0.48 (cm)	0.5245
Height before surgery	4.05 ± 0.59 (cm)	4.05 ± 0.36 (cm)	–
Height after surgery	4.09 ± 0.51 (cm)	4.21 ± 0.56 (cm)	0.4560
Bohler’s angle before surgery	1.60 ± 25.94 °	1.63 ± 13.55°	–
Bohler’s angle after surgery	19.97 ± 9.48°	21.70 ± 10.33°	0.5611
Gissane’s angle before surgery	118.57 ± 12.21°	122.10 ± 14.95°	–
Gissane’s angle after surgery	116.51 ± 10.61°	120.18 ± 16.76°	0.3789
Varus (+) or valgus (−) before surgery	1.81 ± 4.20°	3.30 ± 5.17°	–
Varus (+) or valgus (−) after surgery	1.52 ± 2.35°	1.41 ± 1.59°	0.8571

The average surgery times were 87.00 ± 9.78 min for the open reduction internal fixation group and 85.86 ± 11.17 min for the closed reduction percutaneous fixation group (*P* = 0.614). There was no significant difference between the two groups.

### Clinical outcome results

The patients were followed up for an average of 16.53 ± 3.95 months (range, 12 to 27). According to the AOFAS ankle-hindfoot score, the open reduction plate fixation group score was 80.29 ± 6.15 (range 67–91), and that of the closed reduction percutaneous group was 83.62 ± 6.95 (range 74–100) (*t* = 1.73, *P* = 0.0957 > 0.05). The visual analog score of the traditional open reduction plate fixation group was 1.50 ± 1.22 (range 0–4), whereas that of the percutaneous fixation group was 0.81 ± 0.87 (range 0–3) (*t* = 2.159, *P* = 0.0364 < 0.05). The length of stay of the traditional open reduction plate fixation group was 9.63 ± 2.72 days (range 6–16), whereas that of the percutaneous fixation group was 6.71 ± 1.85 days (range 4–10) (*t* = 4.141, *P* = 0.0002 < 0.05). The complication rate of the open reduction plate fixation group was 20.8% (5/24) (wound infection in 1 foot, skin necrosis in 1 foot, chronic pain in 3 feet), and that of the closed reduction percutaneous fixation group was 4.8% (1/24) (chronic pain in 1 foot) (*P* = 0.0051). The clinical outcome results are shown in Table 3.

**Table 3:** Outcome assessments

	ORIF	CRPF	*P*
AOFAS hindfoot score	80.29 ± 6.15	83.62 ± 6.95	*P* = 0.0957
VAS postoperatively	1.50 ± 1.22	0.81 ± 0.87	*P* = 0.0364 *
Complication rate	20.8%	4.8%	*P* = 0.0051 *
Length of hospital stay	9.63 ± 2.72 days	6.71 ± 1.85 days	*P* < 0.001 *

*P < 0.05

During the 1-year follow-up, 1 patient developed traumatic arthritis and received subtalar fusion in the open reduction group, which was Sanders type IV. No hardware was removed.

## Discussion

The standard treatment of displaced intra-articular calcaneus fracture is open reduction plate fixation. Open reduction plate fixation can provide better fracture visualization and facilitate direct restoration of the subtalar joint and the anatomic parameters, including length, width, height, and alignment [16]. Although open reduction plate fixation may offer superior reduction and rigid fixation, it has some disadvantages, such as skin necrosis and infection complications, which occur in approximately 20–37% of such cases [17–21], with higher rates among smokers and diabetics.

In recent years, many scholars have reported minimally invasive plate fixation with sinus tarsi incision or small lateral wall incisions and external fixation for calcaneus fracture [11–13]. The minimally invasive treatments have become prevalent as a way to reduce tissue damage and the complication rate and to accelerate healing [8, 22–24]. Compared with open reduction internal fixation, the timing of surgery is more liberal because surgery may be performed soon after the injury. However, minimally invasive percutaneous fixation relies on indirect K-wire reduction techniques that require substantial use of fluoroscopy to ensure exact anatomic reduction, which remains an important issue [25]. Additionally, many surgeons have questioned the quality of reductions that surgeons are achieving with minimally invasive techniques, and very few comparative studies of open reduction plate fixation versus closed reduction percutaneous fixation have reported the reduction results and functional outcomes.

To overcome the disadvantage of potentially suboptimal reduction in minimally invasive surgery, we devised two types of traction device based on that of Fröhlich et al. [26]. We analyzed 40 cases including 45 feet with calcaneus fractures to determine whether the open reduction plate fixation and closed reduction percutaneous fixation groups might sustain the same reduction effects and followed up with the patients to determine the clinical outcomes.

Many traction devices exist for calcaneus fracture reduction [27]. We believe that our bilateral traction device is superior to some types of unilateral traction. The traction device may retract the gap of the subtalar joint to restore the length and height of the calcaneus and to prepare for the width reduction. The width may be reduced by compression on both sides of the calcaneus after traction. Additionally, the alignment (varus and valgus) of the calcaneus may be adjusted easily through bilateral traction in the axial view. Another crucial surgical technique is articular reduction through a bone tunnel. The bone tunnel is made through the cannulated screw from posterior to inferior of the depressed articular surface. Through the created bone tunnel, the central fragment may easily be elevated by a curved smooth periosteal elevator after traction. Bohler’s angle and the joint articular surface can be reduced clearly by the lateral view.

At the same time, our device can also be combined with sinus tarsi incision or small lateral wall incisions. It is reusable and costless and can achieve traction only through K-wires. The traction device is easily assembled and is adjustable for length and varus and valgus angles. This hands-free traction device exists as two types, i.e., 2- and 3-point traction devices. For Sanders type II or III fractures, 2-point traction might be used for direct traction on the subtalar joint. For some Sanders type IV or more comminuted fractures, which may be too comminuted such that 2-point traction is not suitable, 3-point traction can be used for traction on the tibia-calcaneus and calcaneocuboid joint to restore the anatomical shape of the calcaneus. With the aid of the traction device, the proper length, height, and width reductions may be achieved. The articular surface and Bohler’s and Gissane’s angles may be restored under fluoroscopy.

Furthermore, we found that Gissane’s angle did not reflect the displacement of calcaneus fractures on the lateral view. Ultimately, we believe that a satisfactory reduction is crucial regardless of whether open or closed reduction is used to provide a superior functional outcome. Standards for reduction should not be reduced because minimally invasive surgery is chosen. The closed reduction with traction device method may provide a satisfactory reduction effect compared with open reduction.

Many minimally invasive methods of calcaneus fixation exist [11–13, 28]. Our percutaneous fixation does not violate the plantar surface, which may cause additional damage. We used 4.0-mm cannulated screws with shim fixation for the articular surface and 7.0-mm headless screws with injectable calcium phosphate cement for the axis of the calcaneus. The percutaneous screw might restore the calcaneal biomechanics [29], and the injectable calcium phosphate cement might effectively fill the bone defect and increase the initial stability. In our percutaneous fixation group, the clinical outcome results were superior to those of the open reduction plate fixation group. The minimally invasive incision, shorter length of stay, lower VAS score, and a lower complication rate reflected the superior short-term clinical outcomes. However, in the long-term follow-up, the AOFAS scores did not differ significantly.

There were some limitations to this study. First, the number of cases was limited, particularly for Sanders IV fractures. This study describes the early experience and results of closed reduction percutaneous fixation. Second, the outcomes were only evaluated by simple X-rays; CT scan results would be expected to evaluate the quality of fracture reduction in further studies. Third, the follow-up time was relatively brief; follow-up CT scans might provide additional study advantages. Finally, a multicenter randomized comparative study was not available, which might have led to selection bias.

## Conclusion

This study was focused on the comparison of reduction and functional outcomes of minimally invasive surgery using a traction device for open versus closed reduction plate fixation. The traction devices were quite helpful for the closed reduction, and most of the displaced intra-articular calcaneus fractures were satisfactorily reduced. With the use of this device, there were no significant differences in the reduction regarding length, width, height, and Bohler’s, Gissane’s, and varus and valgus angles between the open reduction plate fixation group and closed reduction percutaneous fixation group. The functional outcome assessments showed that the closed reduction percutaneous fixation group had lower VAS scores after surgery, a lower complication rate, and a shorter hospital stay; however, no significant differences in the AOFAS hindfoot scores were observed. With the development of a reduction device, the closed reduction percutaneous fixation method will be not only a viable treatment option but will also have wider indications, which will produce better outcomes in the near future. However, multicenter controlled randomized clinical trials are still required prior to widespread practical implementation.

## Abbreviations

VAS: Visual analog score
AOFAS: American Orthopaedic Foot and Ankle Society
